# Classical Morphology of Plants as an Elementary Instance of Classical Invariant Theory

**DOI:** 10.1371/journal.pone.0006969

**Published:** 2009-09-11

**Authors:** Evgeny V. Mavrodiev

**Affiliations:** Department of Biology, University of Florida, Gainesville, Florida, United States of America; University of Nottingham, United Kingdom

## Abstract

It has long been known that structural chemistry shows an intriguing correspondence with Classical Invariant Theory (CIT). Under this view, an algebraic binary form of the degree *n* corresponds to a chemical atom with valence *n* and each physical molecule or ion has an invariant-theoretic counterpart. This theory was developed using the Aronhold symbolical approach and the symbolical processes of convolution/transvection in CIT was characterized as a potential “accurate morphological method”. However, CIT has not been applied to the formal morphology of living organisms. Based on the morphological interpretation of binary form, as well as the process of convolution/transvection, the First and Second Fundamental Theorems of CIT and the Nullforms of CIT, we show how CIT can be applied to the structure of plants, especially when conceptualized as a series of plant metamers (phytomers). We also show that the weight of the covariant/invariant that describes a morphological structure is a criterion of simplicity and, therefore, we argue that this allows us to formulate a parsimonious method of formal morphology. We demonstrate that the “theory of axilar bud” is the simplest treatment of the grass seedling/embryo. Our interpretations also represent Troll's bauplan of the angiosperms, the principle of variable proportions, morphological misfits, the basic types of stem segmentation, and Goethe's principle of metamorphosis in terms of CIT. Binary forms of different degrees might describe any repeated module of plant organisms. As bacteria, invertebrates, and higher vertebrates are all generally shared a metameric morphology, wider implications of the proposed symmetry between CIT and formal morphology of plants are apparent.

## Introduction

Classical invariant theory (CIT) studies the intrinsic or geometrical properties of polynomials, identifying those properties which are unaffected by a change of variables [1], [2]. The mathematical parts of Introduction and all Methods outlined below are based upon [1]–[4].

The simplest example of a polynomial is the binary form. More accurately, the binary form is a homogeneous function of the variables 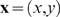, which can be either real or complex:
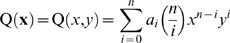
(I)


The integer *n* is the degree of the form.

Under the general transformation of variables: 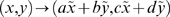,the polynomial (I) is mapped to a new polynomial, given by:
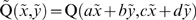
(II)


An **invariant** of the binary form Q(**x**) is a function:
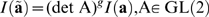
depending on the coefficients of Q, which, up to a determinantal factor (detA), does not change (is invariant) under the action (II) of the general linear group, where:
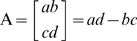
is nonsingular, i.e. an element of some general linear group GL(2).

A **covariant** is a function, depending both on its coefficients and on the independent variables **x** = (*x*, *y*). Therefore: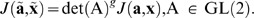 where *g* is the weight of the invariant (or covariant). The degree of *J* is its degree in the independent variables, the order of *J* or *I* is its degree in the coefficients **a** of the equation.

There are several Theorems in CIT. The most important probably are the First and the Second Fundamental Theorems, the Basis Hilbert's Theorem and the Hilbert's “theorem of zeros” (Nullstellensatz).

The First Fundamental Theorem states that every polynomial covariant can be written as a bracket polynomial and the Second Fundamental Theorem states that any identity between bracket polynominals can be deduced from the Three Fundamental Identities. Below I explain both theorems in more detail.

If the invariant or covariant is equal to zero, it vanishes, and we therefore may designate it “trivial”. A binary form 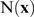 for which all the invariants are trivial is known as a Nullform. In CIT, Hilbert's “theorem of zeros” provides the criteria of the Nullforms.

It has also been proved that if Q(**x**) is a binary form of degree *n*, then there are a finite number of invariants and covariants C_1_ … C_K_ with the property that any other covariant or invariant of Q(**x**) can be written as polynomial of these basic covariants and invariants (Gordan Theorem of Finiteness). Hilbert proved the more general version of Gordan's Theorem (Basis Hilbert's Theorem), and we may say that these basic, “irreducible” or “elementary” invariants and covariants C_1_ … C_K_ are a Hilbert basis of form Q(**x**).

It has long been known that structural chemistry shows an intriguing correspondence with CIT [1]–[3], [5]–[7]. Under this view, the invariant of a system of binary forms (quadratic, cubic etc.) is the analogue of a chemical molecule composed of atoms of various valences: an algebraic binary form of the degree *n* corresponds to a chemical atom with valence *n*. Thus, each physical molecule or ion has an invariant-theoretic counterpart [2], a linear form, for example, corresponds to a hydrogen atom, a quadratic form to an oxygen atom etc. [6]. This theory was developed using the Aronhold symbolical method (see Methods) with a big number of examples [3].

From being a popular mathematical theory at the turn of the 19^th^ and 20^th^ centuries, the CIT gradually lost interest in the opinion of mathematicians after David Hilbert's proof of the main theorem of this theory – the Hilbert's Basis Theorem. As far we can judge, Hilbert never addressed Sylvester's analogy [3], [6]. However, physics have paid serious attention to this idea. For example, Born [8] based the theory of homopolar valences in multiatomic molecules on the analogy of Sylvester [6] as represented in [3] and Weyel with co-authors published at least two papers [9], [10] addressing this “formal, although very appealing mathematical analogy” [9]. Latter, Griffith [11] suggested that in his chemico-algebraic theory, Sylvester [6] had anticipated the essential and central role of a certain type of algebra in the modern theories of chemical valence, and that his theory therefore is a “partial anticipation” of modern quantum chemistry [11], [see also 12].

The basic symbolic process of invariant building in CIT ( “Faltungsprozess” or “Convolution”; see Methods) was characterized as an “*accurate morphological method*” [13] and previously Sylvester called the algebra of the invariants of binary forms as “*Analytical Morphology in its absolute sense*” [14]. However, CIT has never been applied to the morphology of living organisms.

Based on the morphological interpretation of binary form, as well as the process of convolution/transvection of CIT, I show here how CIT can be applied to the structure of plants, especially when conceptualized as a series of plant metamers (phytomers). Classical morphology of plants is not equal to phytonism, but the concept of phytonism is the closest allusion to the basic principle of morphology itself, to Goethe's principle of metamorphosis, according to which one and the same organ make its appearance in multifarious forms [15]. The study of both morphology and plant development, both past and present, has widely accepted phytonism due to its accurate representation of plant form [16]–[29]. We also show, that classical plant morphology is an elementary instance of CIT. To do this, here we provide the morphological interpretation of two general theorems of CIT: the First and Second Fundamental Theorems. We also propose a morphological interpretation of Hilbert's Nullform as a framework for future application of famous Hilbert's “theorem of zeros” to classical morphology.

## Methods

Aronhold's symbolical method proposed, that the binary form 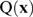 is symbolically the *n-*th power of the linear form (or monomial):
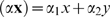
(III)


Therefore we can replace Q(**x**) with a symbolic form:
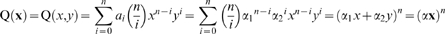


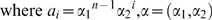
(IV)


Each 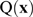 will have a corresponding symbolic form which is essentially found by replacing each coefficient by a symbolic coefficient using equation (IV). We then may call the letter *α* a “symbolic letter” or “symbol” of the coefficients of binary form 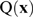.

Equation (III) is also called a “bracket factor of the first kind”.

A “bracket factor of the second kind” is the 2×2 determinant:
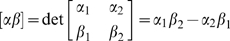
(V)where α_1,2_ and β_1,2_ are symbolic letters.

Using the Aronhold symbolical method we may therefore re-write complicated invariants of 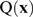 as a simple sequence of bracket factors of the first and second kind. The First Fundamental Theorem of CIT states that if *J*(a, **x**) is a covariant of the binary form (I), then the symbolic form of *J* can always be written as a bracket polynomial P. The weight of the covariant is equal to the number of bracket factors of the second kind in any monomial of P and the degree of the covariant is the number of bracket factors of the first kind of P.

We can associate an atom with bracket factor of the first kind (III) [1]–[3], [5]–[6]. The degree *n* of the factor of the first kind corresponds to the valence of the atom. The connection between two atoms can be described using a bracket factor of the second kind (V)[1]–[3]. For example, the symbolic bracket polynomial 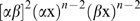 describes a molecule that consists of two atoms: *α* and *β*. Since the bracket factor 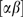 occurs twice, there will be two bonds between atom *α* and atom *β*. So, each bracket factor of the first kind 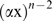 and 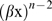 corresponds to *n*–2 free valences of atoms *α* and *β* accordingly [1]–[3].

Also, we may re-write any binary form 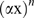 as polar forms: 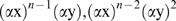 etc. Polar forms are comparable to atoms of non-constant valence [3].

We may symbolically transit two bracket factors of the first kind in a bracket factor of second kind [1]–[3] and call this transition a “Faltungsprozess” [3], or as a “process of convolution (“Faltung”)” [4] or, which is the same in our case [4], as a “transvection” (“Uberschiebung”) of two forms:
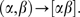
“Convolution” is similar to the process of saturation for of the valences of atoms [1]–[3].

For convolution 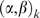 of forms 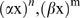, this is true:
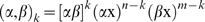
(VI)


If *k* = 1 we may call equation (VI) Jacobian, and if *k* = 2, equation (VI) is a Hessian of two binary forms. For example, an atom of oxygen (valence = 2) corresponds to a binary form of the second degree: 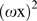. The first and second convolutions of two binary forms 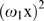 and 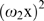 therefore provide an invariant/covariant corresponding to a hypothetical oxygen radical (the trivial covariant Jacobian 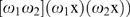 and a stable neutral “molecule” of oxygen (the invariant Hessian 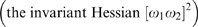


For simplicity we may call a binary form of the second degree a “binary quadratic” (or simply “quadratic”), a binary form of the third degree a “binary cubic” (or simply “cubic”) etc.

## Results and Discussion

### 1. Morphological interpretation of the First Fundamental Theorem of CIT

According to the phytonic theories each organ of a plant (shoot, spikelet, flower etc.) is simply the repetition of phytomer that principally includes the stem joint, the leaf, the axillary bud/meristem, and the secondary roots [17], [20], [21], [23], [25], [27]. We also include in a phytomer the prophylls of the axillary's bud (with hypopodium and mesopodium, if present) [21], [29]. It is not necessary to always associate the bud with the leaf in whose axil it occurs [23], [27], [29], [30].

**Figure 1:**
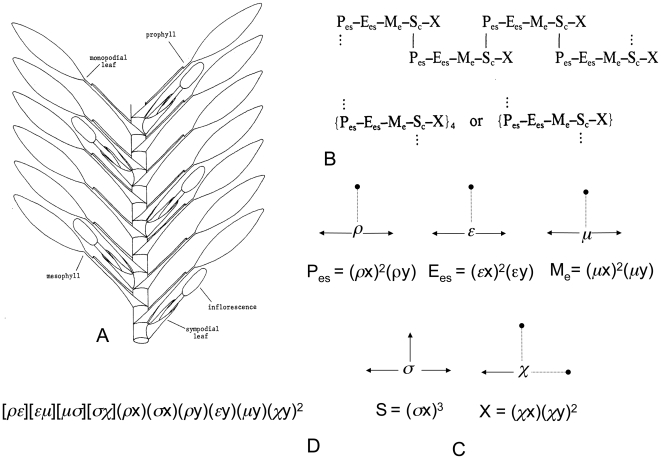
A description of the repeated unit of *Orontium aquaticum* L. (Araceae) containing five phytomers. A Schematic image of *O. aquaticum* [30]. B, Diagram of the repeated unit containing five phytomers [30]. C, A description of B by the covariant of five binary cubics. D, Reducible covariant of five binary cubics corresponded to B. P – prophyll; E – mesophyll; M – monopodial leaf; S – sympodial leaf; X – first phytomer of inflorescence; e – foliage leaf, c – cataphyll, s – sylleptic growth [30]. All morphological terminology and images are from [30].

Because each plant phytomer generally connects with two other neighbours in sequence (Figure 1A and B) and includes the bud, or, in other words, connect with the phytomer of the next order, we propose an analogy: in the simplest case the plant phytomer itself corresponds to a binary cubic (Figure 1C) and a phytomer with a reduced or non-functional axilar bud/meristem corresponds to the polar of a binary cubic (Figure 1C). Binary forms of degrees higher then three can represent a phytomer with serial or collateral buds/meristems. Two connected phytomers can therefore be described by a Jacobian and (or) by a Hessian of two bracket factors of the first kind, for example, of two binary cubics.

Therefore, a shoot or shoot-system built out of a chain of *n* phytomers will always correspond to a covariant of *n* binary forms represented by a symbolic bracket polynomial of bracket factors of the first and second kind (Figure 1D). This provides a morphological interpretation of the First Fundamental Theorem of CIT, according to which the symbolic form of covariant *J*(a, **x**) can always be written as a bracket polynomial P of both kinds of factors [1], [2], [4].

The general morphological sense of the First Fundamental Theorem of CIT therefore is obvious: any morphological structure **always** can formally be expressed in terms of the parts and the connection of these parts. In the context of phytonism, these parts are equal to phytomers.

Irreducible invariants/covariants [1], [2], [4] may correspond to elementary combinations of phytomers (roughly, to “articles” [30], [31]). The invariant/covariant obviously may be reducible even though the biological structure is never found in a reduced form.

### 2. Morphological interpretation of the Second Fundamental Theorem of CIT

According to the Second Fundamental Theorem of CIT, any identity between bracket polynominals can be deduced from the Three Fundamental Identities [1], [2]. If formal morphology is an elementary instance of CIT, all morphological problems will, in principle, resolve using three simple rules, under a morphological interpretation of the Fundamental Identities of CIT.

**Figure 2:**
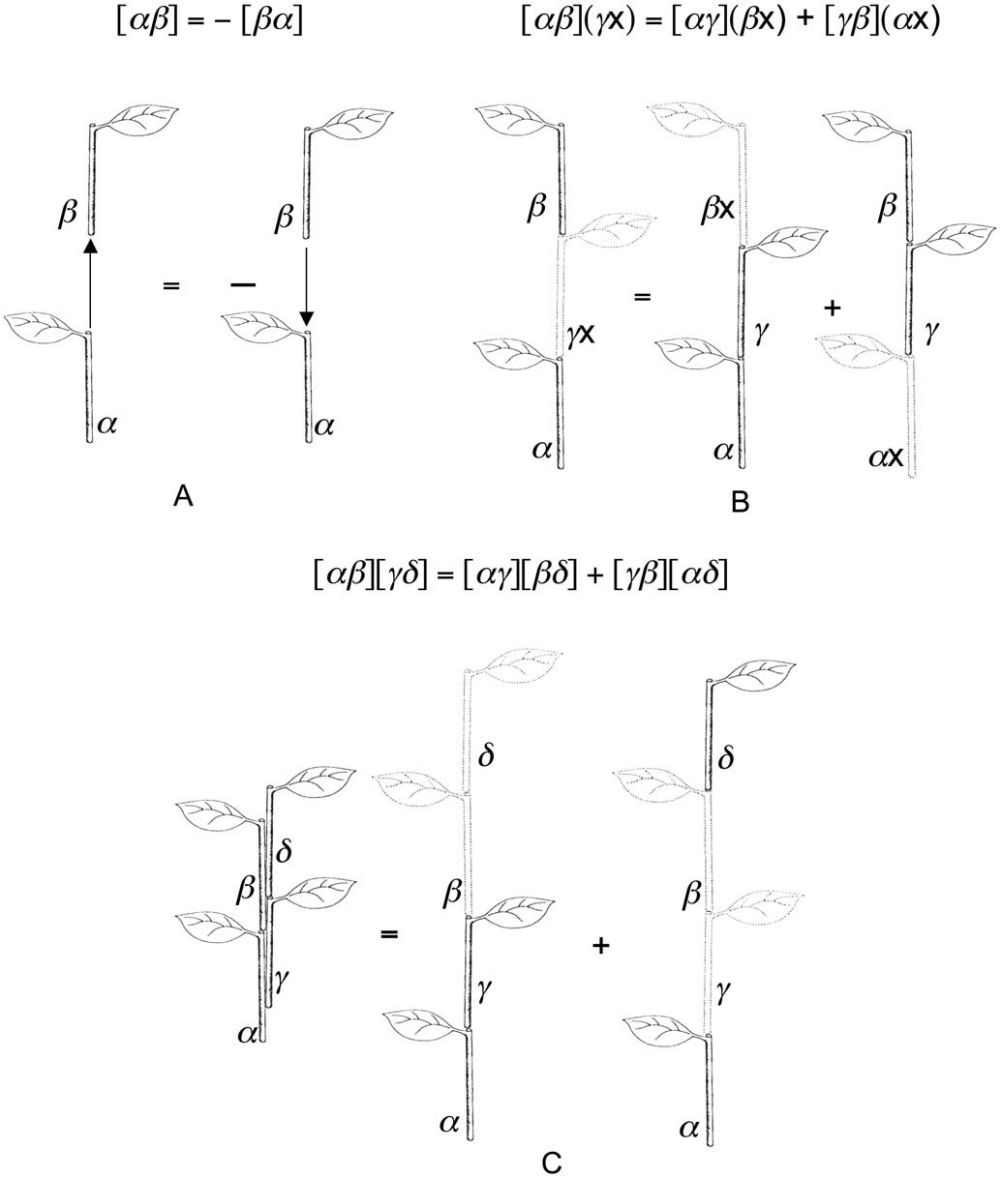
Three Fundamental Identitys of CIT and their morphological interpretation (A–C). The images of phytomers from [31] are re-drawn without buds, reduced phytomers being drawn in a dotted line.

The First Fundamental Identity, 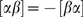, states that the connection of the lowest phytomer with the uppermost one is principally equal to the opposite connection (Figure 2A) [see also 1], [2].

The Second Fundamental Identity, 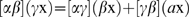, states that in the covariant 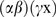, the bracket factor of the second kind 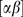 describes the connection between 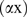 and 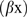, so, if 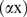 and 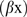 describe two connected entities, then 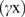, (as well as (*α*x) and 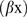 in the same cases), are the reduced entity, located between, above or below the connected entities. In other words, the Second Identity proposes the correct way to visualize a member which has disappeared from the sequence (Figure 2B).

According to Third Fundamental Identity of CIT, 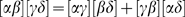. The Second and Third Identities are similar [1], [2], [4] and in the case of so called “monostichous phyllotaxis” [31] it is easy to interpret the Third Identity exactly in the manner of the Second.

**Figure 3:**
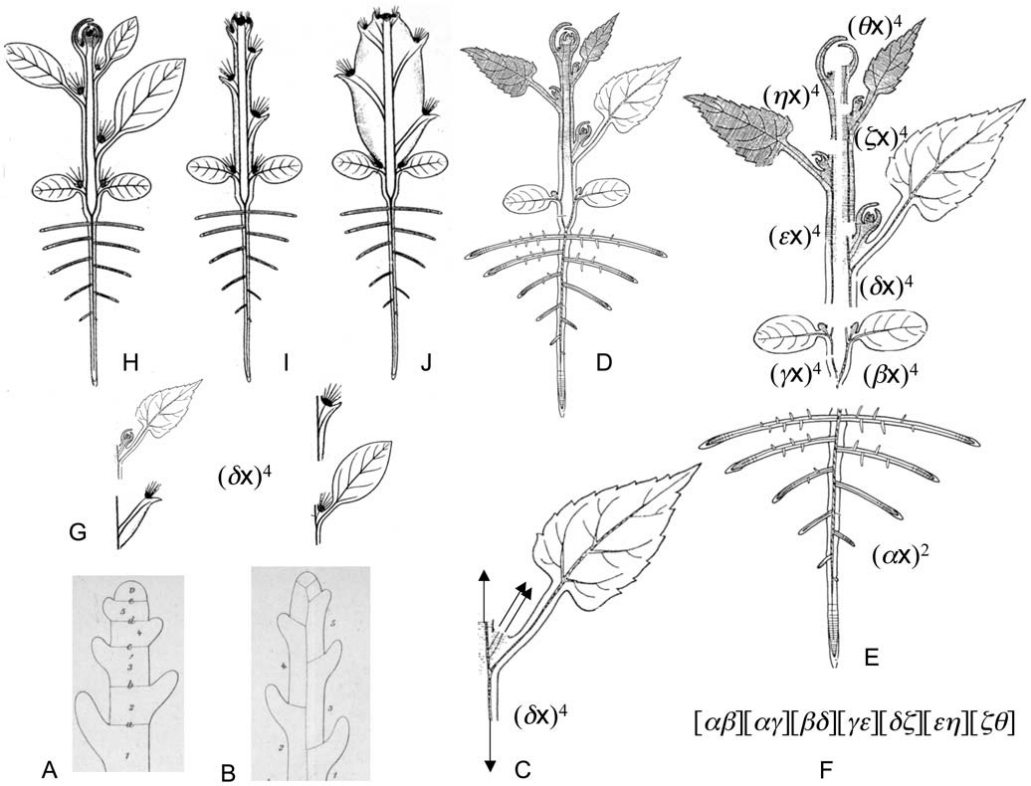
Basic types of segmentation of a stem, descriptions of Troll's bauplan of angiosperms and the principle of variable proportions. All images A–E and G–J are re-drawn from [17] and [32] with or without modifications. A, Holocyclic stem [17] constructed from a series of phytomers each occupying the entire diameter of the axis. B, Mericyclic stem [17] constructed from a series of phytomers each occupying only a portion of the whole diameter of the axis. C, The phytomer of mericyclic stem and the corresponded binary quartic. The prophylls, the hypopodium and the mesopodium are not shown. D, The bauplan of the angiosperm plant (“Urpflanze”) [32]. E, Same as D, but built up from the primary root and shoot with mericyclic stem, each phytomer is drawn with the corresponding binary quartic, primary root drawn with the corresponding binary quadratic. F, Brief notation [1] of covariant corresponded to D, E, and H–J. G–J, The principle of variable proportions [32]: the same binary form corresponds to the phytomers themselves or to the parts of the primary thickening and modified shoot axis corresponding to phytomers, that share the same position in bauplan D but generally differ with the shape and/or proportions (G). The phytomers or corresponded parts of a shoot axis are drawn with prophylls/spines [18], [32] and buds/areoles [18], [32]. Each plant H (“*Pereskia*”, Cactaceace), I (“*Cylindropuntia*”, Cactaceae), and J (“*Ferocactus*”, Cactaceae), as well as the bauplan D therefore may be described by a covariant of the kind F.

To make a more general interpretation (Figure 2C) clear, we need to consider that mosses, gymnosperms and angiosperms all share two basic types of stems: holocyclic and mericyclic [17], [24] (Figure 3A and B). So, if 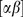 and 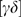 described the connection between corresponded phytomers, then 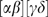 is an exact description of the simple mericyclic stem. The covariant 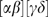 therefore describes the fused pairs of phytomers 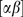 and 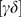, and the covariants 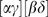 and 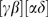 describe the linear sequences of corresponded phytomers, but with reduced pairs 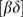 (or 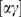) and 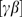 respectively (Figure 2C). The Third Identity therefore generally asserts the principal conformity of the two basic types of stem.

Because the lateral branch of a mericyclic stem is also mericyclic, the simplest description of the phytomer of a mericyclic stem is the binary quartic (Figure 3C). In a simplest case mericyclic stems may be therefore described using a covariants of a binary quartics. This provides a description of Troll's bauplan of the angiosperm plant [32], [33] and the principle of variable proportions (Figure 3D–J) [32], [33].

### 3. Morphological misfits, wholeness and the Nullforms of CIT

According to Goethe, the plant body can be shown as the metamorphosis of the “Leaf “ (“Blatt”) [15]. Using CIT we may re-formulate this observation: Goethe's “Blatt” corresponds to 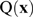 (or, generally, to system 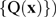 and, therefore, phytomers 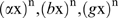 etc are seen as symbolical representations of 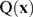 Because a phytomer encloses all the basic organs, we may also treat 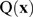 as an entity corresponding to the whole plant, or therefore we may understand the phytomers (*a*x)^n^, (*b*x)^n^, (*g*x)^n^ etc. as a parts that formally represent this whole [34].

If a plant is a single whole, then it does not make sense to treat the structure of this plant in terms of a sequence of phytomers. In the context of the current approach it means that all invariants/covariants of the form 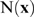 corresponding to this plant, are equal to zero. A binary form 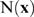 for which all the invariants vanish is known as a Nullform [2]. Certain morphological misfits [31], [35]–[37] are therefore semantically identical to the Nullforms of CIT. The shoot system of *Utricularia*'s species (Magnoliophyta, Lentibulariaceae) is probably, the best-known example of this kind [36], [37]. *Bryopsis corticulans* (Chlorophyta, Bryopsidaceae) [38] is another example.

### 4. Elementary examples of the application of CIT to plant morphology

As shown above, the Third Identity of CIT generally asserts the principal conformity of two basic types of a stem – mericyclic and holocyclic. However due to the interpretation of the Third Identity of CIT, just the description of the mericyclic stem in notation of CIT (Figure 2C) shows that the mericyclic stem cannot be derived from the holocyclic simply by vertical congenital fusion, as Celakovsky suggested [17]. The notation of CIT itself therefore provides not only a compressed manner to describe plants, but sometimes is a way to understand the logic of the form. Another example is a Nullform that, as suggested above, helps us to understand the holistic approaches in plant morphology as a part of a more general theory.

#### a. General remark: the weight of invariant/covariant and morphological simplicity

Since Aristotle simplicity is widely considered as the *sigillum veri* in science [39], especially in the case of physic-chemical disciplines closely connected with math [40], [41]. It is obvious, however, that in classical biology the simplicity postulate may also be a convenient instrument of method [40]. It is also widely believed that simplicity is a reliable guideline for judging the elegance of proofs [41], but like all aesthetic principles, such a criterion may be subjective [39], [41]. The problem of simplicity is therefore of central importance of the epistemology of the natural sciences [39] but the concept of simplicity requires objective formulation [39], [41]. This has unfortunately never been undertaken in the particular case of the morphology of living organisms.

If we describe a morphological structure in the notation of CIT, we can obviously treat the weight of the covariant/invariant that we used as a measure of the simplicity of the description. In other words, we can correspond to each combination of parts of a morphological structure not only an algebraic invariant/covariant but also some natural number, which is the weight of invariant/covariant, the measure of its simplicity. CIT therefore helps us not to discern, but more to express the most parsimonious explanation we are looking for. Thus, if we have several alternative morphological treatments of the same structure, we can select the covariant/invariant with the minimum weight and reject the alternatives.

#### b. A simple example: the double-needles of the umbrella pine

The morphology of needles of *Sciadopitys* (the umbrella pine, Sciadopityaceae), which appear to be born in the axils of scale leaves, is controversial [34], [42], [43]. According to the one point of view, the double-needle of *Sciadopitys* is a pair of fused leaves born on a minimal shoot, axillary to the scale leaf [reviewed in 34], [42], [43]. According to the other view, the scale-leaf and double-needle together are equivalent to one phyllome. In this case, the needle represents the paired basal segments, which arise from the scale-leaf and are fused in front of it [reviewed in 34].

From the shoot morphology of *Sciadopitys* [42] it is obvious that the stem of this plant is mericyclic. If the needle represents the minimum axilar shoot with a merycyclic stem, to describe the needle we need at least three quartics. Again, the quartic 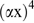 correspond to the phytomer with the scale leaf, the quartics 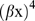 and 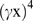 corresponds to the phytomers of the axial shoot. The covariant 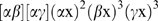 of weight two therefore describe the double needle of the umbrella pine.

But if the double needle of the umbrella pine is equal to one phyllome we need only one quartic 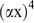 to describe the whole needle. This quartic describes the phytomer with the appendicular part divided into scale and the paired basal segments. So we have to conclude that this treatment is more simple than the alternative one.

Both alternative treatments, however, are questionable from the pure morphological standpoint. If the needle of umbrella pine is a shoot, where are the prophylls of this shoot? In addition, Carrier [reviewed in 43] described the monstrous *Sciadopitys* needle where the slightly bifid character of the extremity of the ordinary needle had become very pronounced, and where a bud was developing on the interval between the two points [reviewed in 43]. If the needle of *Sciadopitys* is one phyllome, how do we explain this unusual morphology of mutants?

This observation of Carrier [reviewed in 43] appears to be strong evidence that the needles of *Sciadopitys* represented deeply modified lateral shoots (“phylloid shoots”, “phylloclades”, “cladodes” etc.), perhaps with reduced prophylls. In this case the double-needle is actually composed of stem derivates.

However, if we treat the needle of the umbrella pine simple as a pair of fused transversal prophylls of the bud that are born in the axil of the scale, then we may associate these prophylls with the same scale-leaf phytomer and again describe the whole double needle of *Sciadopitys* only by single quartic (*α*x)^4^. This solution is close to Engelmann and Mohl's [reviewed in 43] solutions and to the modern concept of a double-needle [42].

If the double-needle of *Sciadopitys* is a pair of fused lateral prophylls, the bud observed by Carrier [reviewed in 43] represents the apex of the axial shoot, which is normally non-developed.

So, there are two simplest treatments of the morphology of needles of *Sciadopitys*, but only one of them explains the data concerning the *Sciadopitys* mutants. Note that without of the description of the needle of *Sciadopitys* in the notation of CIT, it would not be obvious that the two treatments are equal in terms of simplicity.

#### c. The axis with appendages or the chain of the phytomers?

**Figure 4:**
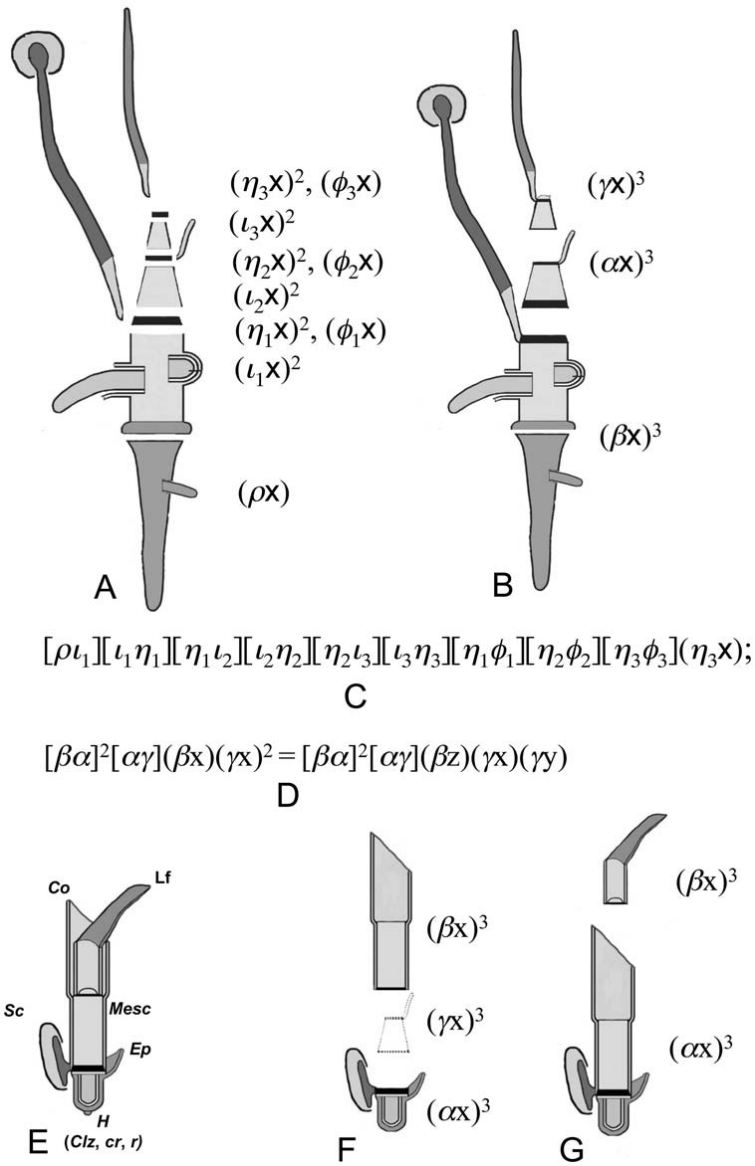
Description of the bauplan of monocot seedling and grass embryo by covariants of different binary forms. A, The image of the bauplan of a monocot seedling from [44], built from primary root, stem, and leaves; (*ρ*x) – linear binary form corresponding to primary root, (*ι*_j_x)^2^ – quadratics, corresponding to hypocotyl (incl. shoot-born roots, coleorhiza and collar) and internodes; (*η*_j_x)^2^ – quadratics, corresponding to nodes, (*φ*_j_x) – linear binary forms, corresponding to cotyledon (drawn connected with endosperm) and upper leaves. B, The image of the same bauplan [44], but built from three phytomers (drawn with corresponding binary cubics), all roots are secondary under this view. C–D, The description of a seedling by covariants of ten binary forms (C) of degrees 1–2 or by irreducible covariant of three binary cubics (D). E, Image of a grass seedling from [44]; *Co* – coleoptile, *Ep –* epiblast, *Mesc –* mesocotil, *Sc* – scutellum, *H* – hypocotyl (*clz* – coleorhiza, *cr* – collar, and *r* – roots); *Lf* – leaf. F, Composition of grass seedling if the coleoptile is the first leaf of the plumule, the scutellum being the cotyledon, the reduced phytomer being drawn in a dotted line. G, Composition of grass seedling in the case where scutellum and coleoptile together form a single cotyledon. All morphological terminology from [44].

According to the “classical” theory there are three basic plant organs: roots, stems and leaves. Stems are subdivided into nodes and internodes; leaves are only found at the nodes in a lateral position [32], [33] (Figure 4A). Under this view, a monocot seedling [44] can be describe by the following covariant 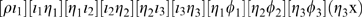 with a weight of nine (Figure 4C). Therefore, to provide a complete treatment we need a simultaneous system [3], [4] of four binary forms.

Let us then make an alternative assumption, that the same seedling is constructed from phytomers (Figure 4B). Under this view, we can describe the seedling using an elementary system of binary cubics, e. g. 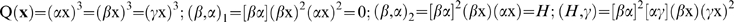 (Figure 4D). The weight of this irreducible covariant is three.

From anatomical and developmental points of view a monopodial shoot with the holocyclic stem may equally be described as a chain of phytomers or as a stem with adherent leaves [45]. Which description is simpler?

If the shoot is a stem with leaves, let us for simplicity exclude nodes and internodes and describe the stem only by one binary form 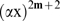. Let us also describe each of m leaves by the linear binary form (*β*x). In this case the covariant 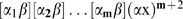 of weight m corresponds to the whole shoot. But if the shoot is a chain of the phytomers 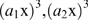, 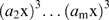, then we can describe it by the covariant 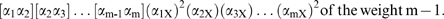



Because in case m >0, m−1<m, the second description is simpler and must be accepted.

#### d. What solution of grass seedling is the simplest one?

One of the most long-lasting and controversial discussions in the field of plant morphology concerns the organ homologies of the grass embryo/seedling, and since the beginning of the 19^th^ century, more than 100 publications have addressed this issue [reviewed in 44], [46].

There are two classical conflicting treatments of the scutellum and coleoptile of the grass embryo: a. the coleoptile is the leaf of the plumule, the scutellum being the cotyledon (Figure 4E and F), or b. the scutellum and coleoptile together form a single cotyledon [44], [46] (Figure 4E and G).

It is not obvious which treatment simpler because in both cases the seedling contain two non-reduced, “physically present” phytomers (Figure 4F and G).

If the scutellum and coleoptile together form a single cotyledon (Figure 4E and G), we may describe the seedling by a Jacobian 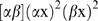
or by the Hessian 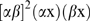. If (*α*x)^3^ and (*β*x)^3^ are equal, the Jacobian 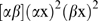 is trivial. If 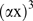 and 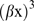 are different, the simplest description of the seedling is the Jacobian 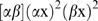.

If the coleoptile is the leaf after the cotyledon, based on the position of the coleoptile and distichous phyllotaxis (½) (diagnostic to Poaceae) we must conclude that the first leaf after the cotyledon in grasses is reduced to nothing (Figure 4F) or to the epiblast, a small scale-like structure with no vascular bundles (Figure 4E). But how does this reduced phytomer affect the complexity of the seedling? To understand this we need a correct way to visualize a member which has disappeared from the sequence. CIT, particularly the Second Identity may help us in this situation.

In the case where we treat the coleoptile as a separate leaf after the scutellum, we must describe the seedling by covariants 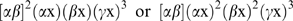. Again if 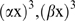, and 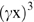 are equal, the covariant 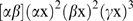 vanishes, but if 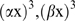, and 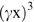 are different, the same covariant is the simplest description of the seedling.

Based on First and Second Identities:
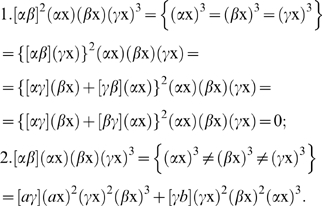



All descriptions of the grass seedling/embryo are summarized in Table 1. Based on this summary we must conclude that the treatment “scutellum + coleoptile  =  cotyledon” is generally more simple. This is in agreement with the current solution of the grass seedling/embryo [44].

**Table 1:** Descriptions of grass seedling/embryo based on the different treatments of coleoptile.

	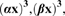 and 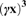 are equal	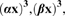 and 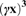 are different
Coleoptile is the leaf of the plumule, the scutellum being the cotyledon	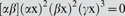 or 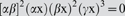	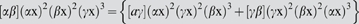
Scutellum and coleoptile together form the single cotyledon	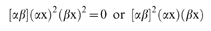	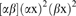

However, if we understand the bud of the grass seedling as a lateral [23], [47]–[50], then the coleoptile is seen to be a single prophyll [23], [47]–[50] of the lateral bud and we may therefore treat the grass seedling as single phytomer [17], [29], describe the seedling only by single cubic 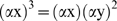 and by this way demonstrate that the corresponding morphological treatment is the most simple one.

#### e. Conclusions and closing comments

CIT can be applied to the structure of plants, especially when conceptualized as a series of plant metamers (phytomers). Whilst in the current study we have concentrated on the relationship between the binary form and the plant phytomer, this is the only one of many examples of the branching and repetition of the morphological and developmental units (cells, meristems, modules etc) that are omnipresent in the plant kingdom [24], [35], [51].

Classical morphology has largely disappeared from scientific discussions in the last ten years or so. Moreover, as Kaplan [33] correctly indicated, the discipline of plant morphology in its pure form has never been widely practiced in the United States. The basic idea of classical morphological approach, as we interpret it, is that there are general, pure mathematical laws of form that are invariant among all organisms, i. e. independent from genetics, embryology and other backgrounds. CIT provides a good opportunity to demonstrate this. Indeed, as bacteria, invertebrates, and higher vertebrates are all generally shared a metameric morphology [52]–[57], much wider implications of the proposed symmetry between CIT and classical morphology of plants are apparent.
